# Infectious Thyroiditis in Children: A Challenging Diagnosis and Management

**DOI:** 10.7759/cureus.103204

**Published:** 2026-02-08

**Authors:** Alexandra Rodrigues, Francisca Guimarães, Joana Capela, Catarina P Mendes, Luís Ribeiro, João Luís Ribeiro de Castro, Teresa Borges, Maria João Oliveira

**Affiliations:** 1 Pediatrics, Unidade Local de Saúde da Guarda, Guarda, PRT; 2 Pediatrics and Neonatology, Unidade Local de Saúde Entre Douro e Vouga, Santa Maria da Feira, PRT; 3 Pediatrics, Unidade Local de Saúde Algarve, Faro, PRT; 4 Pediatric Endocrinology, Centro Materno Infantil do Norte, Unidade Local de Saúde Santo António, Porto, PRT; 5 Endocrinology, Unidade Local de Saúde Nordeste, Bragança, PRT; 6 Pediatric Surgery, Centro Materno Infantil do Norte, Unidade Local de Saúde Santo António, Porto, PRT

**Keywords:** cervical abscess, infectious thyroiditis, pediatrics, pyriform sinus fistula, remnants branchial arch

## Abstract

Infectious thyroiditis is a rare condition, and it is more prevalent in children due to anatomical abnormalities. The main cause is the presence of a pyriform sinus fistula derived from the remnants of the third or fourth branchial arch. This article presents a case of a five-year-old boy with infectious thyroiditis complicated by a cervical abscess. Despite temporary improvement with oral antibiotics, the complaints recurred, and a new course of intravenous antibiotic therapy was necessary. A cervical ultrasound, MRI, and direct laryngoscopy were performed in order to diagnose an anatomical malformation. It was only during surgery that the existence of a fourth branchial arch fistula was observed, complicated by an infected prethyroidal cyst and proximity thyroiditis. After surgery, the patient was discharged and followed up in the pediatric endocrinology consultation, with particular attention to growth, intellectual development, thyroid function, and imaging. This case underscores the importance of thorough imaging of infectious thyroiditis to find out the underlying cause and its surgical resolution to prevent complications and ensure proper thyroid function.

## Introduction

Infectious thyroiditis is a rare condition, and it is predominantly of bacterial etiology [1-3]. It accounts for 0.1-0.7% of all thyroid diseases, and it is more prevalent in children than in adults [2-5]. The thyroid gland is particularly resistant to infection, as it is in an anatomically less accessible area, has a protective capsule, and exhibits good lymphatic and blood drainage, as well as iodine levels that favor its protection [2,4,6]. The main cause of infectious thyroiditis in pediatric age is the presence of a pyriform sinus fistula derived from the remnant at the level of the third or fourth branchial arch. These conditions should be considered, especially when the infection recurs [1,2,4,5]. In such cases, there is also a notably higher prevalence of infection affecting the left lobe of the thyroid gland [1,2,7]. Less frequently, it can also be caused by a thyroglossal duct remnant, patency of the foramen cecum, hematogenous dissemination, anterior esophageal perforation, immunocompromised states, dental abscesses, or endocarditis [1,2,4]. These children present with a sudden onset of neck pain, which is usually unilateral, edema, hyperemia, and tenderness upon palpation of the anterior cervical region with an associated palpable thyroid [1,2-6]. This condition may be accompanied by fever, and it is common to have a recent history of upper airway infection [2,6]. In some cases, cervical discomfort associated with the edema is described, including dysphagia, dyspnea, or dysphonia [1-3]. In this article, we describe the clinical case of a child with infectious thyroiditis, including the investigation, treatment, and follow-up performed.

## Case presentation

A five-year-old male presented to the emergency department with the sudden onset of neck pain and a left cervical mass with inflammatory signs. The previous week, he had acute febrile pharyngitis, which was treated symptomatically. As a pathological personal history, he had nut allergies and intermittent asthma. Immunizations were up to date according to the national vaccination program, including the seasonal influenza vaccine. Given the clinical presentation, a laboratory workup was performed, revealing elevated inflammatory markers (leukocytes 11,580/µL, neutrophils 7,156/µL (61.8%), lymphocytes 3,045/µL (26.3%), C-reactive protein 173.9 mg/L) and hyperthyroidism (thyroid-stimulating hormone (TSH) 0.02 mUI/L (reference value: 0.35-4.94) and free thyroxine (fT4) 23.5 ng/L (reference value: 7-14.8)) (Table 1).

**Table 1:** Laboratory parameters at baseline and day 10 of antibiotic therapy.

Parameter	Reference value	Day 0	Day 10
Leukocytes (/µL)	5,000-13,000	11,580	13,030
Neutrophils (/µL)	1,800-8,000	7,156 (61.8%)	6,593 (50.6%)
Lymphocytes (/µL)	1,200-6,000	3,045 (26.3%)	5,225 (40.1%)
C-reactive protein (mg/L)	<5.00	173.9	7.43
Thyroid-stimulating hormone – TSH (mUI/L)	0.35-4.94	0.02	0.27
Free thyroxine - fT4 (ng/L)	7-14.8	23.5	11.2
Total triiodothyronine - tT3 (ng/mL)	0.6-2.0	-	1.3

**Figure 1:**
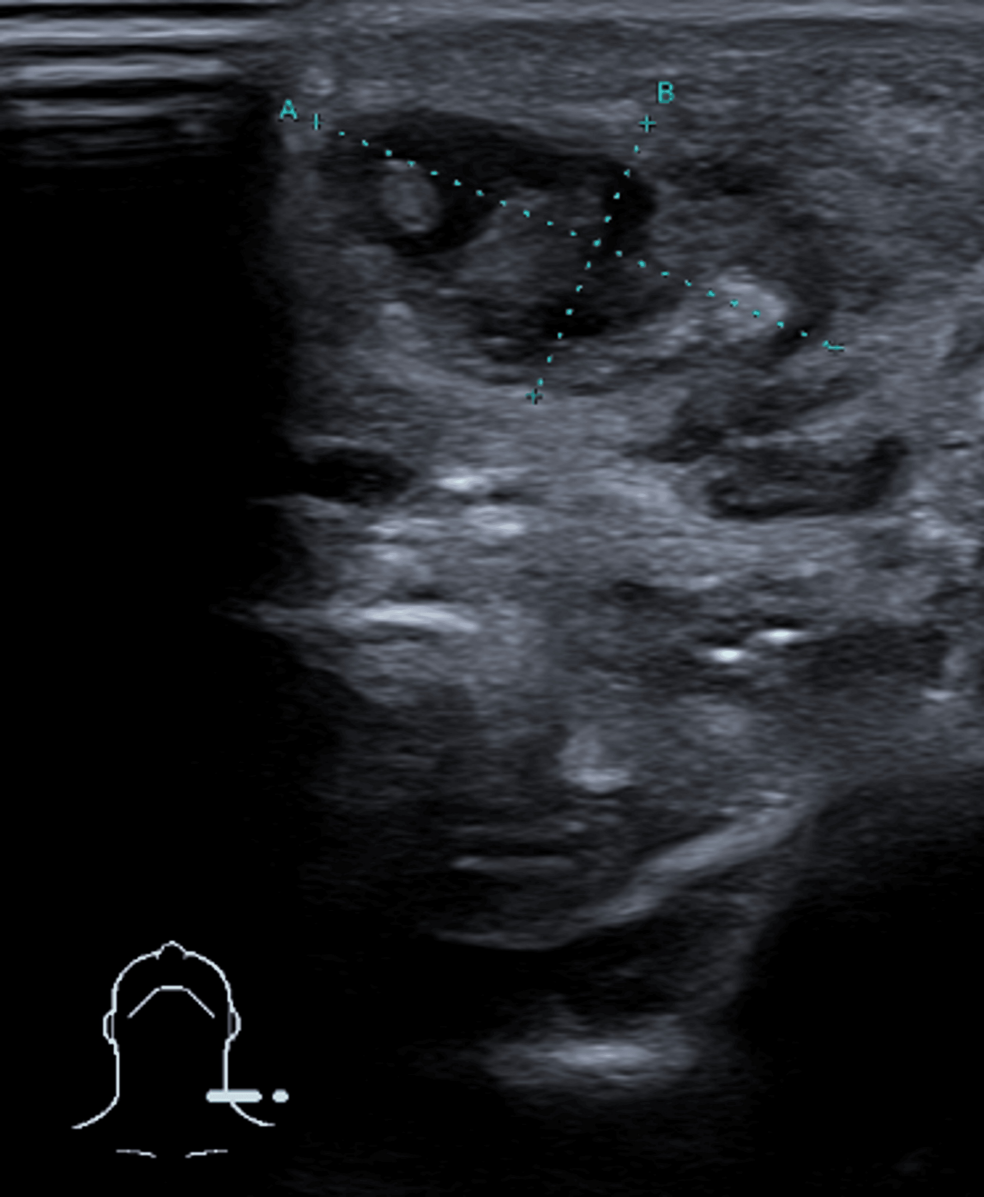
Thyroid ultrasound showing a cervical abscess (20×11×21 mm) in close contact with the left thyroid lobe and isthmus.

**Figure 2:**
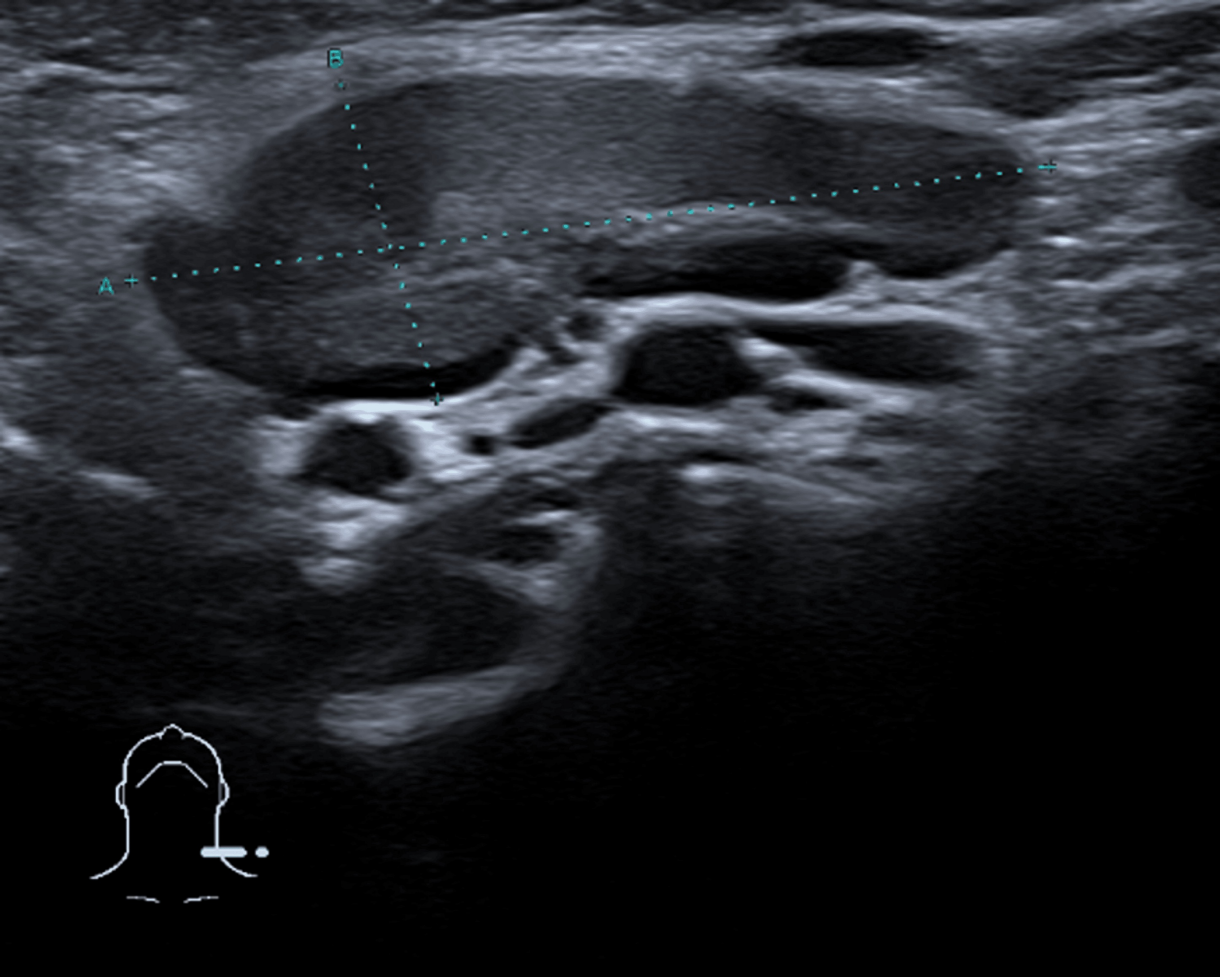
Thyroid ultrasound demonstrating a reactive left submandibular lymph node measuring 36x13 mm.

The thyroid ultrasound demonstrated a globular and heterogeneous appearance, predominantly involving the left lobe, as well as an underlying cervical abscess measuring 20×11×21 mm in close contact with the left hemithyroid and the isthmus, which were displaced posteriorly due to mass effect (Figure 1). Multiple ipsilateral reactive cervical lymph nodes were also noted, the largest located at the left submandibular level, measuring 36×13 mm (Figure 2).

Initial management was performed on an outpatient basis with oral amoxicillin-clavulanic acid (7:1 formulation) at a dosage of 50 mg/kg/day, administered every eight hours for 10 days, in association with ibuprofen every eight hours for the first three days and subsequently as needed. The patient was reassessed on days 3 and 10 of antibiotic therapy, showing gradual clinical improvement, a marked reduction in inflammatory parameters (analytical study at the tenth day of antibiotics showed C-reactive protein 7.43 mg/L), near normalization of thyroid function tests: fT4 11.2 ng/L; total triiodothyronine (tT3) 1.3 ng/mL (reference value: 0.6-2.0); TSH 0.27 mIU/L) (Table 1), and no ultrasonographic evidence of complications. However, 48 hours after completion of antibiotic therapy, the patient experienced a clinical relapse requiring hospital admission. Intravenous antibiotic therapy with ceftriaxone (50 mg/kg/day) and clindamycin (20 mg/kg/day) was initiated. A contrast-enhanced computed tomography (CT) scan was performed to exclude involvement of the posterior and deep cervical spaces, and an ultrasound-guided cervical aspiration was carried out for microbiological identification. Culture results revealed the presence of *Streptococcus constellatus* and anaerobic bacteria, *Finegoldia magna*, *Parvimonas micra*, and *Prevotella buccae*, all of which were sensitive to the instituted antibiotic regimen. This therapy was maintained for a total of 15 days, with progressive clinical and analytical improvement (with reduction of the inflammatory parameters). On the 13th day of treatment, a new ultrasound-guided cervical puncture was performed, yielding a small amount of serous content, which was later found to be sterile in microbiological studies. The patient was discharged home with oral clindamycin for an additional 14 days.

Outpatient follow-up was maintained with clinical monitoring, and a cervical ultrasound was performed the day after completing oral antibiotic therapy. The ultrasound demonstrated persistent heterogeneity of the left thyroid lobe. Anteriorly, a poorly defined hypoechoic area (14×6 mm) was identified at the site of the previous collection, showing residual features. A cervical MRI was also requested to elucidate the etiology of infectious thyroiditis. However, two weeks after stopping the oral antibiotic therapy, clinical recurrence occurred with erythema, edema, and spontaneous discharge of a small amount of purulent content from the same location. The patient was readmitted on intravenous ceftriaxone at 50 mg/kg/day and clindamycin at 20 mg/kg/day.

**Figure 3:**
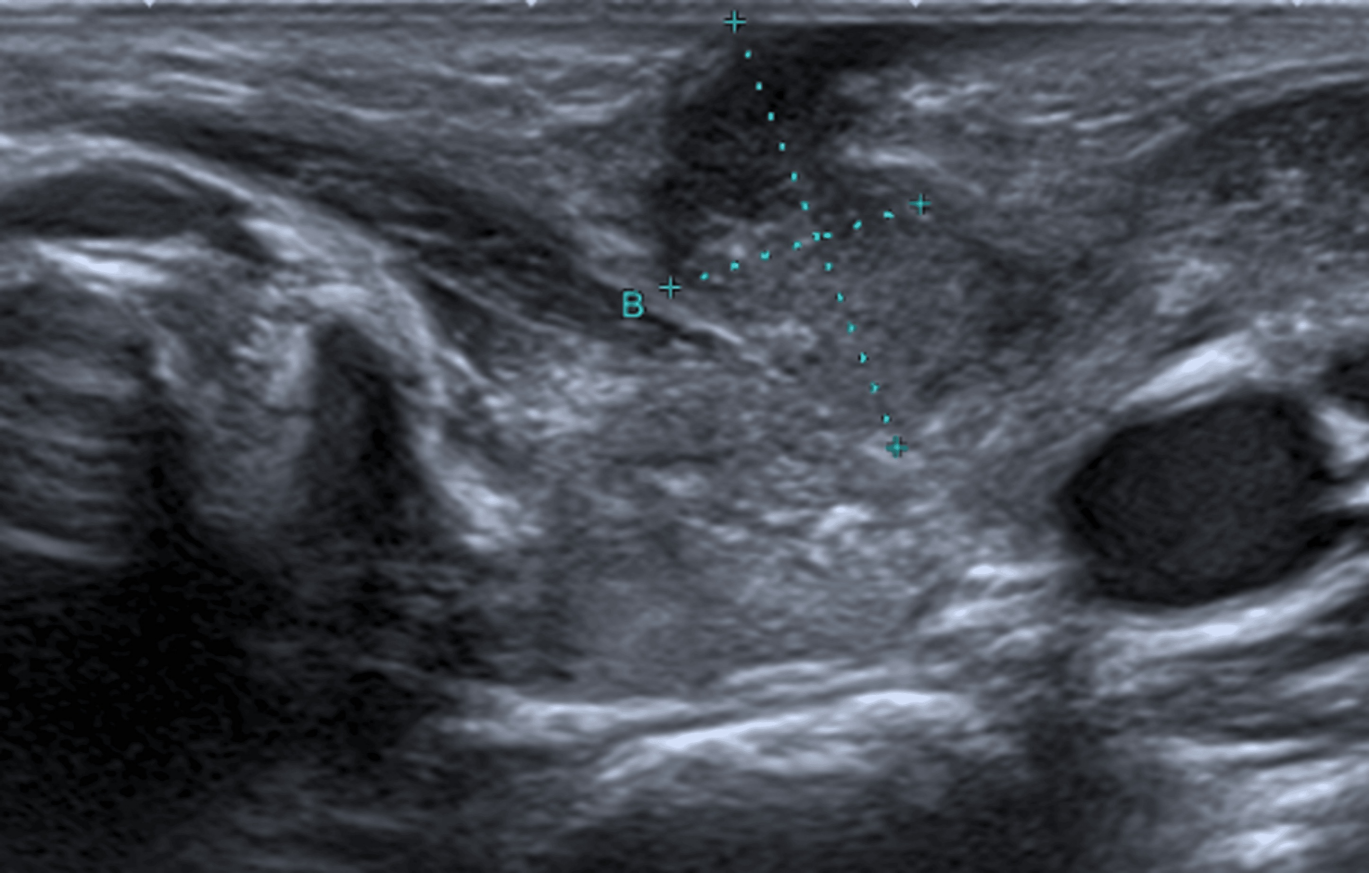
Thyroid ultrasound revealing a tortuous fistulous tract (12×7 mm) with a sinuous course extending toward the skin surface, indicative of external fistulization.

During hospitalization, the thyroid ultrasound revealed a tortuous tract with a sinuous course extending almost to the skin surface (12x7 mm), consistent with fistulization (Figure 3). The tract displayed heterogeneous content with a more liquefied component. Subsequently, the cervical MRI and nasofibroscopy did not visualize any pyriform sinus fistulous tract. Analytical studies detected subclinical hypothyroidism (TSH 6.5 mUI/L and fT4 12.1 ng/L) without other abnormalities. Blood cultures isolated S*treptococcus mitis/oralis,* which was sensitive to the antibiotic prescribed.

On the 18th day of hospitalization, surgical intervention was performed, during which a fistula and the fourth branchial arch cyst were identified and excised in block with the upper half of the left thyroid lobe. The procedure was carried out with careful identification of the left recurrent laryngeal nerve and parathyroid glands, which were preserved. In addition, parathyroid and thyroid functions remained normal, with no transient hypocalcemia or other abnormalities. A cervical drain was removed the day after surgery, and the patient was discharged with oral amoxicillin-clavulanic acid (50 mg/kg/day) for an additional eight days.

After discharge, follow-up was maintained in a pediatric endocrinology consultation to monitor thyroid function and phospho-calcium metabolism, which remained normal throughout. Since the surgical intervention, there have been no further infectious recurrences, and the patient is currently asymptomatic with appropriate growth and good academic performance.

## Discussion

According to the literature, infectious thyroiditis accounts for approximately 0.1 to 0.7% of all thyroid pathologies [2-4], and it is more prevalent in pediatric patients due to its primary association with anatomical abnormalities. The pyriform sinus fistula is the main etiology. However, other causes, such as thyroglossal duct remnant, patency of the foramen cecum, or immunocompromised states, may also be present [1,2,4,8].

In the clinical case described in this article, recurrent infectious thyroiditis was caused by the presence of a pyriform sinus fistula originating from a remnant of the fourth branchial arch. According to bibliographic sources, this etiology accounts for 45% of all infectious thyroiditis cases [1].

The most challenging differential diagnosis for infectious thyroiditis is subacute thyroiditis (also known as De Quervain's thyroiditis) [2,5,6,8]. Both conditions clinically present with pain and inflammatory signs of the thyroid/cervical region and may have associated fever [5,6,8]. While infectious thyroiditis is caused by a bacterial infection, subacute thyroiditis has a viral etiology [5-8]. Moreover, infectious thyroiditis is more prevalent in pediatric patients, whereas subacute thyroiditis is more common in adults [8]. The primary way to distinguish between the two conditions is through imaging. Subacute thyroiditis typically shows a hypoechoic diffuse thyroid on ultrasound, whereas infectious thyroiditis reveals more localized edema (particularly of the left lobe) associated with liquid or abscess formation [5,6]. Infectious thyroiditis also presents with leukocytosis, neutrophilic predominance, and elevated C-reactive protein, which are not common in subacute thyroiditis [5,6].

The microorganisms usually involved in infectious thyroiditis are part of oropharyngeal flora (*Staphylococcus* and *Streptococcus* species) and/or anaerobic species. Sometimes, polymicrobial infection occurs [2-6].

According to the literature, in cases of infectious thyroiditis, intravenous antibiotic therapy is generally preferred over oral antibiotics as initial treatment for acute bacterial thyroiditis. In the present case, an initial course of oral antibiotic therapy was attempted with a favorable response. However, a clinical relapse occurred a few days after treatment discontinuation, ultimately requiring hospital admission and escalation to intravenous antibiotic therapy.

Surgical drainage is often required when there is an inadequate clinical response to intravenous antibiotics and percutaneous drainage. In our case, the patient’s infection is resolved with intravenous antibiotic therapy combined with percutaneous drainage, without the need for surgical intervention. Surgical intervention was performed due to suspicion of an underlying anatomical anomaly and was only undertaken after the infection had improved. This procedure allowed identification and removal of the fistula, preventing further recurrence [2-4].

Imaging is very important, not just to identify the etiology of infectious thyroiditis, given the high prevalence of anatomical abnormalities, but also to establish the extension of the infection and the involvement of nearby tissues [1-4]. Ultrasound is the first-line imaging modality, as it allows differentiation between subacute thyroiditis, typically characterized by a diffusely heterogeneous appearance and reduced vascularity, and infectious thyroiditis, in which an associated abscess may be present. In addition, ultrasound is widely available, cost-effective, and free of ionizing radiation [1-4]. In contrast, once the diagnosis of infectious thyroiditis is established, cervical CT, preferably with intravenous contrast, should be performed to assess the extent of infection and to identify possible involvement of deep cervical spaces. Thoracic CT is generally not required and should be reserved for patients with a history of immunosuppression, in whom the risk of infectious dissemination is higher. Given that infectious thyroiditis in the pediatric population is frequently associated with underlying anatomical anomalies, cervical MRI is necessary for their accurate identification, as it is the most sensitive imaging modality for this purpose. Direct laryngoscopy may be considered when there is a high suspicion of an anatomical anomaly despite negative imaging studies and may even serve as a therapeutic intervention during the acute phase [2,3]. In our clinical case, identification of the underlying anatomical anomaly was only possible in the surgical setting. Due to a high index of suspicion, based on the occurrence of three recurrences and the consistently left-sided localization of thyroiditis, surgical exploration was undertaken, leading to the identification of a pyriform sinus fistula originating from a remnant of the fourth branchial arch with subsequent resolution and a favorable clinical outcome.

Maintenance of follow-up is recommended even after surgical correction of the underlying etiology due to the potential for this condition to induce states of thyrotoxicosis or transient or permanent hypothyroidism [2-4]. Thyrotoxicosis has exaggerated systemic manifestations like hyperpyrexia, tachycardia, severe agitation, confusion, vomiting, and multiorgan dysfunction, and systemic infection is one of the precipitating factors [8].

## Conclusions

In conclusion, infectious thyroiditis is a rare condition. Clinical presentation and physical examination are key elements in its diagnosis, but they should always be complemented by laboratory evaluation and cervical imaging (ultrasound, CT scan, or MRI). This condition highlights the importance of a multidisciplinary approach involving endocrinologists, radiologists, and surgeons to achieve complete resolution of the infection, prevent recurrence or associated complications, and ensure appropriate monitoring of thyroid and parathyroid function.
